# Associations between the stringency of COVID-19 containment policies and health service disruptions in 10 countries

**DOI:** 10.1186/s12913-023-09363-1

**Published:** 2023-04-12

**Authors:** Tarylee Reddy, Neena R. Kapoor, Shogo Kubota, Svetlana V Doubova, Daisuke Asai, Damen Haile Mariam, Wondimu Ayele, Anagaw Derseh Mebratie, Roody Thermidor, Jaime C. Sapag, Paula Bedregal, Álvaro Passi-Solar, Georgiana Gordon-Strachan, Mahesh Dulal, Dominic Dormenyo Gadeka, Suresh Mehata, Paula Margozzini, Borwornsom Leerapan, Thanitsara Rittiphairoj, Phanuwich Kaewkamjornchai, Adiam Nega, John Koku Awoonor-Williams, Margaret E. Kruk, Catherine Arsenault

**Affiliations:** 1 Biostatistics Research Unit, South African Medical Research Council, Durban, South Africa; 2 School of Mathematics, Statistics and Computer Science, University of KwaZulu Natal, Durban, South Africa; 3 Department of Global Health and Population, Harvard T.H. Chan School of Public Health, Boston, USA; 4 World Health Organization, Vientiane, Lao People’s Democratic Republic, Vientiane, Laos; 5 Epidemiology and Health Services Research Unit CMN Siglo XXI, Mexican Institute of Social Security, Mexico City, Mexico; 6 School of Public Health, College of Health Sciences, Addis Ababa University, Addis Ababa, Ethiopia; 7 Studies and Planning Unit, Ministry of Public Health and Population, Port-Au-Prince, Haiti; 8 Public Health Department, Faculty of Medicine, Pontificia Universidad Católica de Chile, Santiago, Chile; 9 Caribbean Institute for Health Research, University of West Indies, Kingston, Jamaica; 10 Office of the Member of Federal Parliament Gagan Kumar Thapa, Kathmandu, Nepal; 11 School of Public Health, University of Ghana, Accra, Ghana; 12 Ministry of Health, Koshi Province, Biratnagar, Nepal; 13 Faculty of Medicine Ramathibodi Hospital, Mahidol University, Bangkok, Thailand; 14 Department of Global Health, The George Washington University Milken Institute School of Public Health, Washington, USA

**Keywords:** COVID-19 restrictions, Health systems, Health services, Pandemic response, Health system resilience, Health care disruptions

## Abstract

**Background:**

Disruptions in essential health services during the COVID-19 pandemic have been reported in several countries. Yet, patterns in health service disruption according to country responses remain unclear. In this paper, we investigate associations between the stringency of COVID-19 containment policies and disruptions in 31 health services in 10 low- middle- and high-income countries in 2020.

**Methods:**

Using routine health information systems and administrative data from 10 countries (Chile, Ethiopia, Ghana, Haiti, Lao People’s Democratic Republic, Mexico, Nepal, South Africa, South Korea, and Thailand) we estimated health service disruptions for the period of April to December 2020 by dividing monthly service provision at national levels by the average service provision in the 15 months pre-COVID (January 2019-March 2020). We used the Oxford COVID-19 Government Response Tracker (OxCGRT) index and multi-level linear regression analyses to assess associations between the stringency of restrictions and health service disruptions over nine months. We extended the analysis by examining associations between 11 individual containment or closure policies and health service disruptions. Models were adjusted for COVID caseload, health service category and country GDP and included robust standard errors.

**Findings:**

Chronic disease care was among the most affected services. Regression analyses revealed that a 10% increase in the mean stringency index was associated with a 3.3 percentage-point (95% CI -3.9, -2.7) reduction in relative service volumes. Among individual policies, curfews, and the presence of a state of emergency, had the largest coefficients and were associated with 14.1 (95% CI -19.6, 8.7) and 10.7 (95% CI -12.7, -8.7) percentage-point lower relative service volumes, respectively. In contrast, number of COVID-19 cases in 2020 was not associated with health service disruptions in any model.

**Conclusions:**

Although containment policies were crucial in reducing COVID-19 mortality in many contexts, it is important to consider the indirect effects of these restrictions. Strategies to improve the resilience of health systems should be designed to ensure that populations can continue accessing essential health care despite the presence of containment policies during future infectious disease outbreaks.

**Supplementary Information:**

The online version contains supplementary material available at 10.1186/s12913-023-09363-1.

## Introduction

In response to the COVID-19 pandemic and in an effort to reduce the spread of infections, countries imposed a series of non-pharmaceutical interventions, such as stay-at-home orders, curfews and quarantines. These measures and policies were known as “lockdowns”, or COVID-19 containment or closure policies. By the end of March 2020 more than 100 countries had instituted either a full or partial lockdown [1].

An increasing breadth of literature has shown disruptions in health services during the initial phase of the COVID-19 pandemic with heterogeneity in the degree and duration of these disruptions [2–16]. Nonetheless, studies found no clear pattern in health service disruptions according to pandemic intensity (e.g., number of COVID cases or deaths) or by country characteristics, income, or health system characteristics [2, 13, 14]. Disruptions in health service utilization during the pandemic could be caused by a combination of factors including a decrease in health facility capacity (due to reallocation of staff or other resources to COVID-19 wards, staff getting infected, or staff burnout), patients choosing to defer medical care due to concerns about exposure to the virus, or people’s inability to pay for medical care from loss of income or employment. The barriers imposed by COVID-19 containment policies such as public transportation closures, stay-at-home requirements or restrictions on internal movement, could also result in declines in non-COVID health care utilization [17–19].

Few studies have sought to understand the relation between the stringency of COVID-19 containment policies and the level of health service disruptions. In 18 low- and middle-income countries, a study found a significant relationship between the stringency of COVID-19 mobility restrictions and disruptions in outpatient consultations, child vaccinations, and the fourth antenatal care visit, whereby a standard deviation in the mobility restrictions stringency was associated with a 3.9 percent reduction in outpatient consultation volume on average [16]. In 11 sub-Saharan African countries, the stringency of COVID-19 containment policies was significantly associated with reductions in outpatient visits and inpatient admissions, where a 10 percentage points increase in the stringency index was associated with a 3.1–3.6 percentage points reduction in these two services [14]. Other studies have looked at country or health-service specific relationships between containment policies and service disruptions. In South Africa, significant reductions in child health visits and HIV services were observed during varying levels of lockdowns [10, 15]. In Nepal, significant increases in certain health services were seen after lockdowns were lifted at local levels [20]. While the aforementioned studies have sought to measure the relationship between the stringency of containment policies and changes in health services, they tend to be limited in terms of geographic areas or in the types of health services covered, focusing largely on maternal and child health services.

In this paper, we investigate associations between the stringency of COVID-19 containment policies and disruptions in 31 health services in 10 low- middle- and high-income countries. We further extend this analysis by examining the relative impact of 11 individual containment or closure policies on health service disruptions. This study was undertaken as a part of the QuEST Network, a global partnership of researchers and policy makers conducting health systems research. Participating countries were selected based on prior collaborations. Colleagues from 10 countries with different income levels, health system types, severity of COVID and government responses to COVID joined the effort.

## Methods

### Data sources

Data on the volume of varying health services provided from January 2019 to December 2020 (24 months) were extracted from administrative sources and Routine Health Information Systems (RHIS) in Chile, Ethiopia, Ghana, Haiti, Lao People’s Democratic Republic, Mexico, Nepal, South Africa, South Korea, and Thailand. In Ethiopia, Ghana, Haiti, Lao PDR, Nepal and South Africa, RHIS data were extracted from the DHIS2 platform. The DHIS2 platform is the world’s largest health management information system software used by more than 73 countries [21]. In Chile, Mexico, South Korea and Thailand, the data were obtained from various administrative health datasets including the health information system of the Ministry of Health of Chile and of the Mexican Institute for Social Security (IMSS), the South Korea National Health Insurance Service (NHIS) Health Facility Claims Database, and the National Health Database of the Ministry of Public Health of Thailand, respectively.

In Ghana, Haiti, Nepal, and South Korea the datasets included all health facilities in the country reporting to the DHIS2 [2]. In Ethiopia, the Tigray region was excluded due to the ongoing conflict and lack of reporting in late 2020. In Lao PDR and Thailand only visits from public sector facilities were available and in Mexico only the facilities that are run by IMSS. In Chile, certain indicators included only the public sector. In South Africa, we obtained data from the KwaZulu-Natal Province only and the dataset included all health facilities in that province.

We used a series of data cleaning and validation procedures in countries with disaggregated data (Chile, Ethiopia, Haiti, Laos, Nepal and South Africa). First we identified positive outliers (greater than 3.5 standard deviations from the mean trend) and set any outliers as missing [22]. We did not assess negative outliers since decreases in utilization were expected during the COVID-19 pandemic. For each indicator, we also excluded any health facility with poor reporting completeness (i.e., those that reported less than 15 out of 24 months). In the other four countries, data validation was performed by the data custodians. Further information on these country-specific datasets and cleaning methods were published previously [2].

Data on COVID-19 containment policies were obtained from the Oxford COVID-19 Government Response Tracker (OxCGRT) dataset [1]. Two additional COVID-19 government responses – declaration of a state of emergencies and implementation of curfews – were also collected in all ten countries from online news sources and were validated by local researchers. The number of new COVID-19 cases each month were obtained from the COVID-19 Data Repository of the Center for Systems Science and Engineering (CSSE) at Johns Hopkins University [23]. Gross domestic product (GDP) per capita for each country was obtained from the World Bank World Development Indicators database [24].

### Measures

#### Health service disruptions: relative service volumes

We obtained data on a total of 31 health services covering a broad range of health needs (Supplemental Table 1). Because not every service was measured in every country, we analyzed the health services according to five categories: (1) service use overall and injuries (e.g., total outpatient visits, total inpatient admissions, road traffic injuries), (2) reproductive, maternal, and newborn health (e.g., deliveries, antenatal and postnatal care visits), (3) child health services and vaccinations (e.g., sick child visits, measles vaccinations), (4) antiretroviral therapy (ART) and (5) chronic disease services (e.g., diabetes visits, cancer and tuberculosis screening) (Supplemental Table 1). Monthly service volumes were aggregated at the national level in each country. To capture the level of change in each health service during COVID months (April to December 2020), we calculated *relative service volumes* by dividing monthly service volumes during COVID by the average service volume pre-COVID. The pre-COVID period included January 2019 to March 2020. The resulting outcome of interest represents the monthly service provision relative to the average before the pandemic. A value of 1 on any given month during COVID would indicate that the same number of patients were seen that month compared to the average pre-COVID, while a relative service volume value of 0.1 would indicate that the numbers of patients seen that month is only 10% of that on average pre-COVID.

#### Independent variables

In the main analysis, we used the OxCGRT stringency index as the exposure variable [1]. The OxCGRT stringency index is a composite measure based on nine containment, closure and health system response indicators tracked by the OxCGRT and measured on a daily basis in countries. The nine containment policies are: school closures, workplace closures, public events cancellations, restrictions on gathering, public transport closures, stay-at-home requirements, restrictions on internal movement, restrictions on international travel, and public information campaigns. The index captures the stringency of governments’ responses to the COVID-19 pandemic and uses values ranging from 0 to 100, with 100 being the most stringent [1]. To provide an illustration of the meaning of different stringency index values, Table 1 describes the different policies in place on four specific days in four countries, and the resulting value of the stringency index at that time. In the main analysis, we used the mean stringency index at the national level for each month from April to December 2020. Other independent variables included the total number of new COVID-19 cases at the national level every month from April to December 2020 and the latest estimates of GDP per capita available from the World Bank.

**Table 1:** COVID-19 containment policies in place on four specific days in four countries and resulting OxCGRT stringency index values

**Nepal** April 1, 2020 **Stringency Index: 96.3**	**Chile** May 1, 2020 **Stringency Index: 73.15**	**Ghana** June 1, 2020 **Stringency Index: 56.48**	**Lao PDR** July 1, 2020 **Stringency Index: 20.37**
⇒ All schools closed ⇒ All workplaces closed except for essential workers ⇒ All public events cancelled ⇒ Gatherings restricted to 10 people or less ⇒ Public transports closed ⇒ Stay-at-home requirement with few exceptions ⇒ Movement restricted between regions ⇒ Total border closure ⇒ Coordinated public information campaigns	⇒ All schools closed ⇒ Workplaces closed for some sectors ⇒ All public events cancelled ⇒ Gatherings restricted to 100 people or less ⇒ Stay-at-home requirement with few exceptions ⇒ Movement restricted between regions ⇒ Total border closure ⇒ Coordinated public information campaigns	⇒ All schools closed ⇒ Workplace closure recommended, or open with significant alterations ⇒ All public events cancelled ⇒ Gatherings restricted to 100 people or less ⇒ Total border closure ⇒ Coordinated public information campaigns	⇒ School closures recommended^a^ ⇒ Travelers required to quarantine if arriving from certain countries ⇒ Coordinated public information campaigns

Source: Oxford COVID-19 Government Response Tracker^1^

^a^All policies listed were implemented at the national level for the exception of school closures in Lao PDR which were only recommended in some regions of the country

### Statistical analysis

We used a multi-level (including variables at country and country-month level) linear regression model to estimate the association between the OxCGRT stringency index and health service disruptions across 10 countries.

We used variance inflation factors with a threshold of 4 to examine the presence of multicollinearity (correlation amongst the independent variables) [25]. The model building process commenced with a saturated model including both random intercepts at the country-level and a random-coefficient for stringency as well as interactions between fixed effects [26]. These nested models were compared using the likelihood ratio test, resulting in the final parsimonious model structure below:

$$Y_{it}=\beta_0+\beta_1\left({stringency}_{it}\right)+\beta_2\left({COVID19\;cases}_{it}\right)+\beta_3\left({GDP}_i\right)+\beta_4\left(service\;type\;2\right)+\beta_5(service\;type\;3)+\beta_6\left(service\;type\;4\right)+\beta_7\left(service\;type\;5\right)+\mu_i+\epsilon_{it}$$

Where ${Y}_{it}$ is relative service volume in country *i* during month *t*. *Stringency* is the mean stringency index for country *i* during month *t*. *COVID-19* cases is the total number of new COVID-19 cases in country* i* during month *t*. GDP_i_ is GDP per capita in country *i*. *Service type 2 to 5* are the health service category dummies (service use overall and injuries, child health services, ART services, or chronic disease services, with the reference category being service type 1 (reproductive, maternal and newborn health services). ${\mu }_{i}$ is a country-level random intercept and ${\epsilon }_{it}$ is a normally distributed error term. Models also used robust standard errors. To facilitate the interpretation of model coefficients, the stringency index was rescaled to a factor of 10, and GDP per capita to a factor of 1000 (ie. these variables were multiplied by the relevant factors and this form of the variable was included in the model).

To assess the robustness of the effect of stringency on relative service volumes we repeated the analyses using other forms of the main exposure variable: 1) the median stringency index per month (instead of the mean) and 2) using the maximum stringency index observed in a month. These models were fitted using the same fixed and random effects structure as described above. The within-country correlation was estimated using adjusted and unadjusted intracluster correlation.

As a sub-analysis we assessed associations between 11 individual COVID-19 containment policies and the relative service volumes. We included the nine response indicators used in the OxCGRT stringency index and two additional indicators: presence of curfews and state of emergency in place. The nine response indicators from OxCGRT stringency index were recoded as binary variables for ease of interpretation (Supplemental Table 2). Each of the 11 containment policies were added separately to regression models with the same fixed and random effects structure as described above (excluding the stringency index).

## Results

Our analysis included data from ten countries including three in Latin America and Caribbean, three in Sub-Saharan Africa, three in East Asia and the Pacific and one in South Asia (Table 2). GDP per capita ranged from $ USD 2,297 in Ethiopia to $ USD 42,251 in South Korea. The average number of new diagnosed COVID-19 cases per million population per month in 2020 varied widely from 0.48 in Lao PDR to more than 3,500 in Chile (Table 2). Chile had the highest average monthly stringency index during the study period (79.70), while Lao PDR had the lowest (44.53).

**Table 2:**
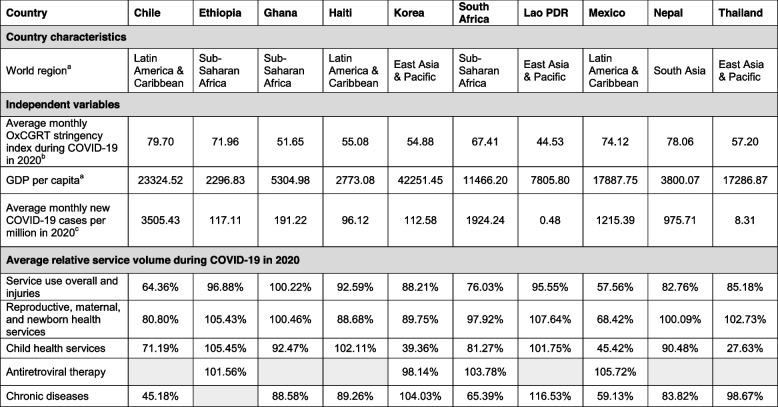
Country characteristics and average disruptions in health services during COVID-19 in 2020

Cells in grey indicate that data on the service category was not available in the country. Relative service volumes were obtained by dividing the monthly number of services provided at the national level by the average monthly volume provided during the pre-COVID-19 period (January 2019 to March 2020). The result, expressed as a percentage, represent the monthly service provision relative to the average before the pandemic

^a^World regions and GDP per capita are from the World Bank World Development Indicators [24, 27]

^b^The Oxford COVID-19 Government Response Tracker (OxCGRT) index was obtained from https://github.com/OxCGRT/covid-policy-tracker

^c^April to December 2020, Source: Center for Systems Science and Engineering at Johns Hopkins University [23]

Our final dataset included a total of 1,467 relative service volume observations estimated monthly for each health service from April to December 2020. Countries with the largest health service disruptions included Chile, Mexico, and South Africa. For example, in Chile, the number of consultations for chronic disease services from April to December 2020 was only 45% of that provided on average pre-COVID-19. Ethiopia and South Korea were among the least affected countries. Average monthly relative service volumes by categories of services during the COVID-19 period are provided in Table 2. Chronic disease care (including visits for hypertension and diabetes and cancer and tuberculosis screening) and child health services (e.g., visits for children with diarrhea or pneumonia) were among the most affected services. Reproductive, maternal, and newborn health services (e.g., delivery care and c-sections) were generally less affected. The magnitude of service disruptions estimated using an interrupted time series (ITS) analysis was described in a previous study [2].

**Fig. 1:**
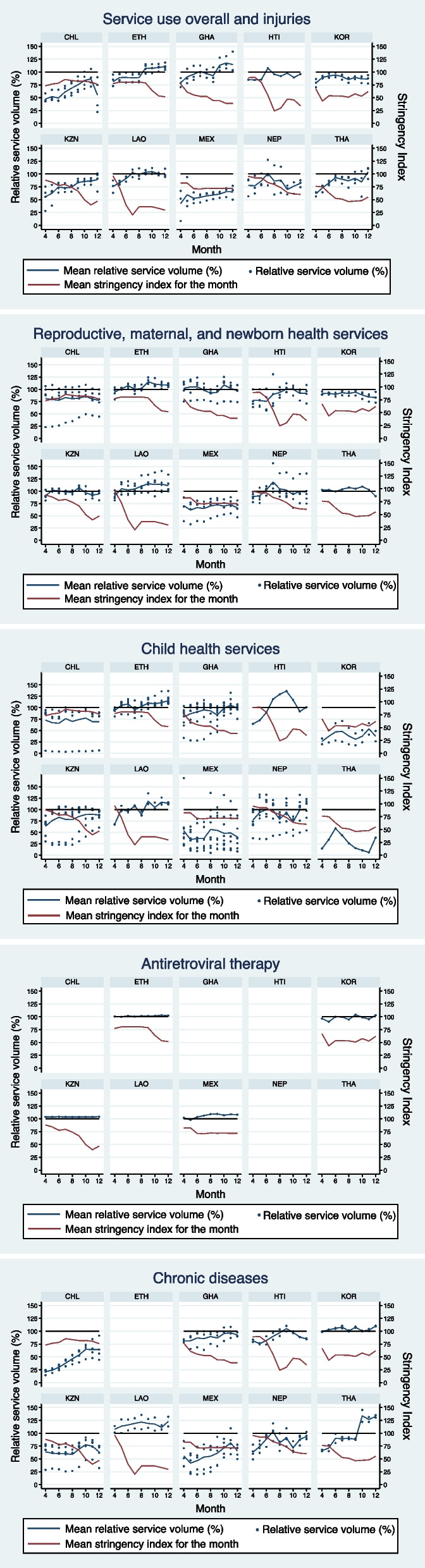
Trends in relative service volumes and the OxCGRT stringency index from April to December 2020. The post-COVID months of April (month 4) through December (month 12) 2020 are shown on the x-axes. The left y-axis is the relative service volume (%) and the right y-axis is the OxCGRT stringency index. The red line is the mean stringency index over these nine months. The navy dots represent the relative service volume (%) for individual health services each month (listed in Supplemental Table 1). The navy line is the mean relative service volume (%) per service type over time. The black line is a reference line for 100% relative service volume and 100% stringency index. CHL is Chile, ETH is Ethiopia, GHA is Ghana, HTI is Haiti, KOR is South Korea, KZN is KwaZulu-Natal Province, LAO is Lao People’s Democratic Republic, MEX is Mexico, NEP is Nepal and THA is Thailand

The relationship between the stringency index and relative service volumes is shown in Fig. 1. Several health services were considerably lower than the average pre-COVID (where the blue line is below 100% in Fig. 1). The mean stringency index (shown as a red line in Fig. 1) was relatively constant from April to December 2020 in Chile, Mexico, and South Korea. In contrast, it varied considerably in the other countries, often starting with high stringency in April and May 2020 which was slowly reduced over time.

Results from multi-level linear regression models are presented in Table 3. After adjusting for COVID cases per million, health service type and GDP per capita, a 10% increase in the mean stringency index was associated with a 3.3 percentage point (95% CI -3.9; -2.7) reduction in relative service volume. COVID-19 cases per million were not associated with relative service volumes. A higher GDP per capita was also associated with lower relative service volumes. We also found relatively high within-country correlation evidenced by a covariate-adjusted intracluster correlation coefficient (ICC) of 0.17 (0.07–0.34).

**Table 3:** Results from multi-level linear regression model for the association between the OxCGRT stringency index and relative service volumes

	**Estimate**	**95% CI**	***p*** **-value**
**Mean stringency index (per 10 percent)**	-3.342	-3.942, -2.741	< 0.001
**COVID-19 cases per million population**	-0.001	-0.0026, 0.0002	0.087
**Service type**			
Reproductive, maternal and newborn health services	Reference	N/A	N/A
Service use overall and injuries	-10.021	-14.970, -5.072	< 0.001
Child health services	-11.927	-19.109, -4.744	0.001
Antiretroviral therapy	13.318	-4.404, 31.040	0.141
Chronic disease services	-10.841	-22.742, 1.061	0.074
**GDP per capita (per 1000 unit)**	-0.581	-1.111, -0.051	0.032
**Variance estimates**			
Country-level random effects	89.283		
Residual	442.885		

Mean stringency index (${\beta }_{1}$) is the mean stringency index value for the month in each country, rescaled to a factor of 10. COVID-19 cases (${\beta }_{2}$) is the COVID-19 cases per million monthly in each country. Service type categories (${\beta }_{3}$) for service volume included: (1) reproductive, maternal, or newborn, (2) service use overall and injuries, (3) child health services, (4) antiretroviral therapy and (5) chronic diseases. GDP (${\beta }_{4}$) is GDP per capita, rescaled to a factor of 1000

In the adjusted model, two health service types (service use overall and injuries and child health services) were associated with significantly lower relative service volumes (worst disruptions) compared to reproductive and maternal services. Associations between the stringency index and relative service volumes was robust to alternate specifications including using the monthly median stringency index and the monthly maximum stringency index instead of the mean (see supplemental Tables 3 and 4).

**Fig. 2:**
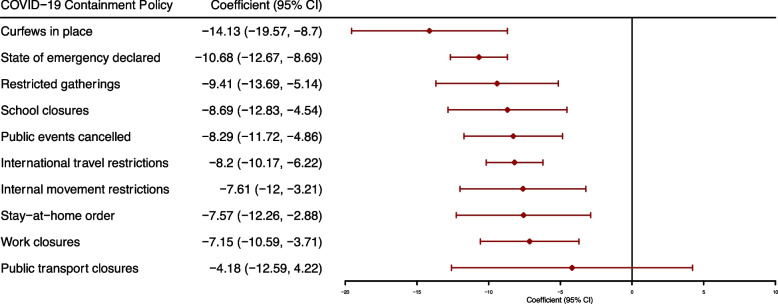
Results from multi-level effects linear regression models for the association between individual COVID-19 containment policies and relative service volumes. Associations between individual COVID-19 containment and closure policies were assessed. Each of the 11 containment policies were added separately to regression models with same fixed and random effects structure described. The model included COVID-19 cases as the COVID-19 cases per million monthly in each country, service type categories for service volume (1) reproductive, maternal, or newborn, (2) service use overall and injuries, (3) child health services, (4) antiretroviral therapy and (5) chronic diseases), GDP as GDP per capita, rescaled to a factor of 1000. The *public information campaigns* policy, collected by OxCGRT, was excluded as the policy was always in place in all countries over the study period

Associations between each of the 11 individual COVID-19 containment or closure policies and relative service volumes were assessed by fitting separate multilevel regression models, all of which included a country random effect and the following independent variables: COVID-19 cases per million, health service type and GDP per capita. We found that each containment policy was negatively associated with relative service volumes. Coefficients ranged from a 4.2 percentage-points decline in relative service volumes for public transport closures to a 14.1 percentage-point decline for curfews. Curfews, restrictions on gatherings and presence of a national state of emergency had the largest coefficients (Fig. 2). These three policies were associated with a 10-percentage point or greater reduction in relative service volumes.

## Discussion

In this paper, we used RHIS and administrative data to estimate the associations between the stringency of COVID-19 containment policies and health service disruptions in 10 countries in 2020. We found that a 10% increase in the mean stringency index was associated with a 3.3 percentage-point reduction in relative service volumes after adjusting for COVID caseload, health service type and country GDP. We also found that nearly all individual containment policies were also associated with significantly lower essential health service use, with curfews and presence of a state of emergency being associated with the greatest disruption in service utilization. In contrast, COVID-19 cases were not associated with health service use. Although the overall effect size we find is small (3.3 percentage-point reduction with 10% increase in stringency), this finding has policy relevance and demonstrates a lack of health system resilience. Any reduction in essential health service use could have important consequences on health, particularly in lower income countries where health care utilization had low baseline levels. In future pandemics, policy makers must ensure that the population can continue accessing essential care while policies are in place to contain outbreaks.

Our study highlights that utilization of essential health services during a pandemic is affected by the types of policies implemented by governments to respond to the crisis. There are many mechanisms by which COVID-19 containment and closure policies could affect people’s willingness or ability to seek health services. Although essential services were permitted during stay-at-home orders, people might still be deterred from seeking care if restrictions were rigidly enforced. In South Africa, people reported fearing being arrested or fined as a reason for not visiting healthcare facilities in April 2020 [28]. In Nepal, strict lockdowns were enforced by the police and citizens were punished if they defied restrictions [29]. In addition, the majority of individuals in the countries studied rely on public transport systems. In 2020, public transports were either fully interrupted, passenger capacity was limited, or they were restricted to short timeframes for transport of essential workers. In some countries, the cost of public transport also increased in 2020 [30–32]. Stringent restrictions might also increase people’s fear of infection in general and when visiting health facilities [19, 33, 34]. Furthermore, the economic effects of COVID lockdowns were distributed inequitably [11, 35, 36]. These lockdowns and containment policies had significant economic impacts on many people, especially those working in informal sectors [37]. Periods of reduced or no income may be associated with lower health care seeking in countries with high out-of-pocket costs. Nonetheless, restrictions may have also led to a reduced need for certain health services including fewer road traffic injuries from reduced mobility and fewer communicable diseases from social distancing, handwashing and mask wearing.

It is important to note that COVID caseloads during this period were not associated with health service disruptions. A study in 18 low- and middle-income countries also found no significant relationship between COVID-19-related deaths and the magnitude of change in any health service [16]. During the first wave of the pandemic, many countries put in place stringent restrictions even when caseloads were low as understandably, much was still unknown about COVID-19 and its effects [37]. For example, in Lao PDR, the worst health service disruptions took place in April 2020, when less than 20 total COVID-19 cases had been recorded in the country.

It is apparent that COVID-19 containment policies have had important indirect effects beyond infection control. COVID-19 restrictions had deleterious economic effects on people globally, especially women [35, 36]. Reports of violence against women and children also increased during COVID-19 lockdowns [38, 39]. Studies have also found that lockdowns may have worsened chronic disease and mental health outcomes. In Nepal, worsening health outcomes among older adults with pre-existing conditions have been reported in part due to significant difficulties in obtaining medications during lockdowns [40]. In Chile, important indirect effects on cardiovascular mortality were reported and were found to be inequitably distributed between women and men [41]. In a study across 15 countries, more stringent COVID containment policies were associated with more psychological distress [42]. COVID-19 restrictions have also resulted in a loss of quality of life. A study using online quality of life surveys estimated that 3,259 million quality-adjusted life-years have been lost globally due to COVID restrictions [43].

Our study shows that the stringency of containment policies was also linked to a reduction in health service utilization. Our findings are consistent with those of other studies that have assessed lockdowns and health service utilization [10, 15]. To our knowledge, only two other multi-country studies have looked at associations between the stringency of COVID-19 restrictions and health service disruptions [14, 16]. Our study contributes to this literature and expands the range of health services and countries studied thus far. We included a broad range of health services and data from 10 countries with varying income levels, COVID caseloads and types of pandemic response.

Nonetheless, our study has limitations. Although a range of countries were included, the association between the stringency of COVID containment policies and service volumes might not be generalizable to all countries or contexts. Second, the associations we describe cannot be interpreted as causal. Models were adjusted for GDP per capita, monthly number of new COVID-19 cases, and type of health service. Nonetheless, it is possible that other variables ( e.g., quality of governance or population age or health needs) may confound the association between restrictions and health services. Third, the administrative datasets used in Chile, Mexico, South Korea and Thailand may differ from the DHIS2 data used in the other six countries, However, co-authors ensured that indicator definitions were comparable across countries. In addition, in some countries, routine health data excluded private facilities, telemedicine visits or some regions in the country [2]. It is also possible that the reporting of routine health data was affected by the pandemic, but rigorous cleaning and verification methods were used and only facilities continuously reporting throughout the study period were included [2]. In addition, COVID cases depend on countries’ surveillance systems and testing capacities and may have been under-counted due to limited testing capacity in the early months of the pandemic, which might bias our results. Although the containment policy tracking followed rigorous validation methods, it is possible that the policies and policy dates could be misclassified [1]. Furthermore, while the association between the stringency of containment policies and disruptions in specific health types (e.g., antiretroviral therapy) may be of interest, our study did not have sufficient power to conduct these stratified analyses by service type. Finally, our study includes data until December 2020. Associations between the stringency of policies and health service utilization has likely evolved in later months of the pandemic.

Our study contributes to understanding the effect of pandemic responses on health service utilization [14, 16]. Although COVID containment policies were crucial to reducing COVID-19 mortality in many contexts, it is important to consider the indirect effects of these restrictions. These indirect effects will likely have long-term population health consequences, as many of the countries studied were already struggling to meet healthcare needs before the pandemic. Containment policies or closures might be necessary to reduce infection, but they should be combined with other policies to promote essential health service utilization. Policies to promote essential health services during times of crisis should be expansive and extend beyond just maternal and child health [44]. Several service adaptations to maintain essential non-COVID healthcare have been implemented in the studied countries and have included the use of telemedicine, community outreach and innovations for medicine delivery. In South Africa, the central Chronic Medicines Dispensing and Distribution program was expanded for community delivery of drugs for chronic diseases [45]. Similarly, in Thailand, service adaptions included telemedicine, shipping of medicine for chronic disease patients and home delivery of medicine by village health volunteers [46–48]. In Chile, an online platform was developed by the Ministry of Health to provide mental health care [49]. In Ethiopia, adaptations to RMNCH services included an increase in phone based follow-up care as well as provision of multiple months of contraceptives at a single visit [50]. Multi-sectoral collaborations and private partnerships were also designed to support the Ethiopian health system [51]. Multi-month dispensing of antiretroviral therapy (ART) guidelines were also distributed by The United States President's Emergency Plan for AIDS Relief (PEPFAR) in many countries [52]. In Nepal, various hospitals and the Nepal Medical Association initiated telemedicine services and deferred elective surgeries and procedures so that resources could be reallocated to other services [53]. The Ministry of Health in Mexico launched home visits to detect COVID-19 cases while also delivering health promotion and prevention activities, including continuity of care for patients with uncontrolled chronic diseases [54]. These adaptations should be standardized and expanded in times of crisis while ensuring quality of care when services are adapted.

## Conclusion

Recently, the WHO revealed that an estimated 14.9 million excess deaths occurred globally between January 2020 and December 2021 of which only 5.42 million were reported COVID deaths, calling for more resilient health systems globally [55]. Health service disruptions likely contributed to this increase in indirect mortality. Plans for pandemic response should consider health system resilience in order to minimize the indirect health consequences of pandemics. As policy makers weigh re-implementing containment policies to reduce infection spread during future waves of COVID or future pandemics, it is important to take these indirect effects into consideration. This study reiterates the importance of resilience in health systems and the need for greater adaptive and transformative capacity, which has the potential to lessen the adverse impact of restrictions imposed during future crises [56].

## Supplementary Information

**Additional file 1: Supplemental Table 1.** Health services by service type category in 10 countries. **Supplemental Table 2.** Definition of containment policies and dichotomous recoding. **Supplemental Table 3.** Results from multi-level linear regression model for the association between the OxCGRT stringency index and relative service volumes (median stringency index). **Supplemental Table 4.** Results from multi-level linear regression model for the association between the OxCGRT stringency index and relative service volumes (max stringency index).

## Data Availability

The data used in Chile are publicly available from https://deis.minsal.cl/. The data Thailand are publicly available from the Ministry of Public Health: http://hdcservice.moph.go.th/. The data from the Mexican Institute for Social Security were deposited in a repository [57]. In all other countries, the data are restricted, and permissions to access the data must be obtained from respective Ministries of Health. Data on containment and closure policies are publicly available from the Oxford COVID-19 Government Response Tracker (OxCGRT) repository: https://github.com/OxCGRT/covid-policy-tracker . All statistical codes used for the analysis are publicly available from a GitHub repository: https://github.com/catherine-arsenault/HS-performance-during-covid-do-files.
