# Supplementary material for: Associations between the stringency of COVID-19 containment policies and health service disruptions in 10 countries
Source: BMC Health Serv Res. 2023 Apr 12;23:363. doi: 10.1186/s12913-023-09363-1 (PMC10096103; doi:10.1186/s12913-023-09363-1)
Supplement: Supplementary file 1 — Additional file 1: Supplemental Table 1. Health services by service type category in 10 countries. Supplemental Table 2. Definition of containment policies and dichotomous recoding. Supplemental Table 3. Results from multi-level linear regression model for the association between the OxCGRT stringency index and relative service volumes (median stringency index). Supplemental Table 4. Results from multi-level linear regression model for the association between the OxCGRT stringency index and relative service volumes (max stringency index). [file 12913_2023_9363_MOESM1_ESM.pdf]

Associations between the stringency of COVID-19 containment policies and health service disruptions in 10 countries
Reddy T, Kapoor NR, et al.

Supplemental materials

Supplemental Table 1. Health services by service type category in 10 countries

Service type	Service	CHL	ETH	GHA	HTI	KOR	KZN	LAO	MEX	NEP	THA
Service use overall and injuries	Outpatient visits		X	X	X	X	X	X	X	X	X
	Inpatient admissions	X	X	X		X	X	X	X	X	X
	Road traffic injuries	X	X	X			X	X			X
	Emergency room visits	X	X			X			X	X	
	Trauma admissions						X				
	Surgeries	X									
Reproductive, maternal, and newborn health services	Deliveries	X	X	X	X	X	X	X	X	X	X
	Antenatal care visits	X	X	X	X	X	X	X	X	X	
	Caesarean sections	X	X	X		X	X	X	X	X	
	Family planning visits	X	X	X	X			X	X	X	
	Postnatal care visits	X	X	X	X		X	X		X	
Child health services	Sick visits for malaria			X							X
	Sick visits for diarrhea		X	X		X	X		X	X	
	Sick visits for pneumonia	X	X	X		X	X		X	X	
	Screened for malnutrition		X						X		
	BCG vaccinations	X	X	X			X	X	X	X	
	Pentavalent vaccinations	X	X	X			X	X	X	X	
	Measles vaccinations	X	X	X			X		X	X	
	Polio (3rd dose) vaccinations		X	X				X	X	X	
	Pneumococcal vaccinations	X	X	X			X	X	X	X	
	Rotavirus vaccinations		X	X			X		X		
	Fully vaccinated by 1		X	X	X		X				

Antiretroviral therapy	People on antiretroviral therapy (ART)		X			X	X		X		
Chronic diseases	Hypertension visits	X		X	X	X		X	X	X	X
	Diabetes visits	X		X	X	X	X	X	X	X	X
	Mental health care	X				X			X		
	Cervical cancer screening						X		X		
	Breast cancer screening	X							X		
	TB screening						X				
	TB case detection			X			X			X	
	TB treatment						X				
	HIV tests									X	

These are the services extracted from Health Management Information Systems (HMIS) for 10 countries, organized into service types. Countries are represented with International Organization for Standardization country codes

Supplemental Table 2. Definition of containment policies and dichotomous recoding

COVID-19 Restriction or Containment Policy	Definition	OxCGRT code	Dichotomous recoding (if applicable)
School closing	Record closings of schools and universities	0 - no measures	0
		1 - recommend closing or all schools open with alterations resulting in significant differences compared to non-Covid-19 operations	0
		**2 - require closing (only some levels or categories, eg just high school, or just public schools)**	**1**
		**3 - require closing all levels**	**1**
Workplace closing	Record closings of workplaces	0 - no measures	0
		1 - recommend closing (or recommend work from home) or all businesses open with alterations resulting in significant differences compared to non-Covid-19 operation	0
		**2 - require closing (or work from home) for some sectors or categories of workers**	**1**
		**3 - require closing (or work from home) for all-but-essential workplaces (eg grocery stores, doctors)**	**1**
Cancel public events	Record cancelling public events	0 - no measures	0
		1 - recommend cancelling	0
		**2 - require cancelling**	**1**

Restrictions on gatherings	Record limits on gatherings	0 - no restrictions	0
		1 - restrictions on very large gatherings (the limit is above 1000 people)	0
		**2 - restrictions on gatherings between 101-1000 people**	**1**
		**3 - restrictions on gatherings between 11-100 people**	**1**
		**4 - restrictions on gatherings of 10 people or less**	**1**
Close public transport	Record closing of public transport	0 - no measures	0
		1 - recommend closing (or significantly reduce volume/route/means of transport available)	0
		**2 - require closing (or prohibit most citizens from using it)**	**1**
Stay at home requirements	Record orders to "shelter-in-place" and otherwise confine to the home	0 - no measures	0
		1 - recommend not leaving house	0
		**2 - require not leaving house with exceptions for daily exercise, grocery shopping, and 'essential' trips**	**1**
		**3 - require not leaving house with minimal exceptions (eg allowed to leave once a week, or only one person can leave at a time, etc)**	**1**
Restrictions on internal movement	Record restrictions on internal movement between cities/regions	0 - no measures	0
		1 - recommend not to travel between regions/cities	0
		**2 - internal movement restrictions in place**	**1**

International travel controls	Record restrictions on international travel<br><br>Note: this records policy for foreign travelers, not citizens	0 - no restrictions	0
		1 - screening arrivals	0
		**2 - quarantine arrivals from some or all regions**	**1**
		**3 - ban arrivals from some regions**	**1**
		**4 - ban on all regions or total border closure**	**1**
State of Emergency	State of emergency or state of disaster in place country-wide	-	-
Curfew	Restrictions on the times that businesses could be open or that citizens could be outside country-wide	-	-

Source: Oxford COVID-19 Government Response Tracker[1]
All policies were extracted from OxCGRT database except *State of Emergency* and *Curfew*, which were tracked by the research team as binary variables, whether the policy was in place or not. The OxCGRT policies were re-coded as binary variables for interpretability in the model. All OxCGRT policies, except *International Travel Restrictions*, included additional flag data that denoted if the policy was in place nationally at only one or many local levels. For the purposes of this analysis, all OxCGRT policies were only recorded if they were in place at the national level.

Supplemental Table 3: Results from multi-level linear regression model for the association between the OxCGRT stringency index and relative service volumes (median stringency index)

	Estimate (SE)	*95% CI*	*p-value*
**Median Stringency index (per 10 percent)**	-3.121 (0.226)	-3.563;-2.678	<0.001
**Cases per million**	-0.001 (0.0007)	-0.0026;0.0001	0.078
**Service**			
Reproductive, maternal and newborn health services	Reference	N/A	N/A
Service use overall and injuries	-10.024 (2.524)	-14.972;-5.077	<0.001
Child health services	-11.929 (3.663)	-19.109;-4.749	0.001
Antiretroviral therapy	13.315 (9.045)	-4.413;31.042	0.141
Chronic disease services	-10.841 (6.072)	-22.742;1.06	0.074
**GDP (per 1000 unit)**	-0.584 (0.277)	-1.127;-0.042	0.035
**Variance estimates**			
Country-level random effects	92.175 (46.909)		
Residual	442.708 (75.728)		

This table represents the same model as the main model in the paper except the median stringency index in the month is used instead of the mean stringency index. Median stringency index (β_1) is the median stringency index value for the month in each country, rescaled to a factor of 10. COVID-19 cases (β_2) is the COVID-19 cases per million monthly in each country. Service type categories (β_3) for service volume included: (1) reproductive, maternal, or newborn, (2) service use overall and injuries, (3) child health services, (4) antiretroviral therapy and (5) chronic diseases. GDP (β_4)is GDP per capita, rescaled to a factor of 1000.

Supplemental Table 4: Results from multi-level linear regression model for the association between the OxCGRT stringency index and relative service volumes (max stringency index)

	Estimate (SE)	95% CI	p-value
**Max Stringency index (per 10 percent)**	-2.805 (0.309)	-3.412;-2.199	<0.001
**Cases per million**	-0.001 (0.0007)	-0.0025;0.0003	0.133
**Service**			
Reproductive, maternal and newborn health services	Reference	N/A	N/A
Service use overall and injuries	-10.027 (2.521)	-14.969;-5.086	<0.001
Child health services	-11.939 (3.66)	-19.113;-4.766	0.001
Antiretroviral therapy	13.308 (9.048)	-4.425;31.041	0.141
Chronic disease services	-10.839 (6.073)	-22.741;1.063	0.074
**GDP (per 1000 unit)**	-0.569 (0.292)	-1.142;0.004	0.052
**Variance estimates**			
Country-level random effects	98.267 (51.143)		
Residual	446.488 (75.381)		

This table represents the same model as the main model in the paper except the maximum stringency index in the month is used instead of the mean stringency index. Max stringency index (β_1) is the maximum stringency index value for the month in each country, rescaled to a factor of 10. COVID-19 cases (β_2) is the COVID-19 cases per million monthly in each country. Service type categories (β_3) for service volume included: (1) reproductive, maternal, or newborn, (2) service use overall and injuries, (3) child health services, (4) antiretroviral therapy and (5) chronic diseases. GDP (β_4)is GDP per capita, rescaled to a factor of 1000.
